# Reciprocating intestinal flows enhance glucose uptake in *C. elegans*

**DOI:** 10.1038/s41598-022-18968-1

**Published:** 2022-09-21

**Authors:** Yuki Suzuki, Kenji Kikuchi, Keiko Numayama-Tsuruta, Takuji Ishikawa

**Affiliations:** 1 Graduate School of Engineering, Department of Finemechanics, Tohoku University, 6-6-01 Aramaki, Aoba, Sendai, Miyagi 980-8579 Japan; 2 Graduate School of Biomedical Engineering, Tohoku University, 6-6-01 Aramaki, Aoba, Sendai, Miyagi 980-8579 Japan

**Keywords:** Bioenergetics, Small intestine, Biophysics, Muscle contraction, Biomedical engineering, Animal behaviour, Animal physiology

## Abstract

Despite its physiological and pathological importance, the mechanical relationship between glucose uptake in the intestine and intestinal flows is unclear. In the intestine of the nematode *Caenorhabditis elegans*, the defecation motor program (DMP) causes reciprocating intestinal flows. Although the DMP is frequently activated in the intestines, its physiological function is unknown. We evaluated the mechanical signature of enhanced glucose uptake by the DMP in worms. Glucose uptake tended to increase with increasing flow velocity during the DMP because of mechanical mixing and transport. However, the increase in input energy required for the DMP was low compared with the calorie intake. The findings suggest that animals with gastrointestinal motility exploit the reciprocating intestinal flows caused by peristalsis to promote nutrient absorption by intestinal cells.

## Introduction

Nutrient absorption is indispensable for animal survival and offspring prosperity. In most animals, the intestine is the major site for digestion of ingested food and nutrient absorption^1,2^; it is one of the oldest organs in the history of evolution. The intestinal functions of digesting food and absorbing nutrients are driven by intestinal peristalsis, which is mediated by periodic muscular contractions and relaxations^3^. Experimental studies and numerical simulations have revealed relationships among intestinal movement, intestinal flow, and mixing of gut contents^4–20^. For example, peristalsis in the intestine of zebrafish larva contributes to transport and mixing of the gut contents^4,5^. Higher intestinal flow rates and larger intestinal volumes enhance the absorption of nutrients such as glucose, vitamins, and minerals in rat intestine in vivo^6–12^. Experiments involving human participants, rats, and pigs in vivo, as well as rat and pig intestines in vitro, have shown that glucose uptake and insulin secretion after feeding decrease as the viscosity of gut contents increases^13–20^. However, the relationships between intestinal movements and nutrient uptake in the intestine are unclear because of difficulties in the visualization and reproduction of nutrient absorption in the intestines during digestion.

**Figure 1:**
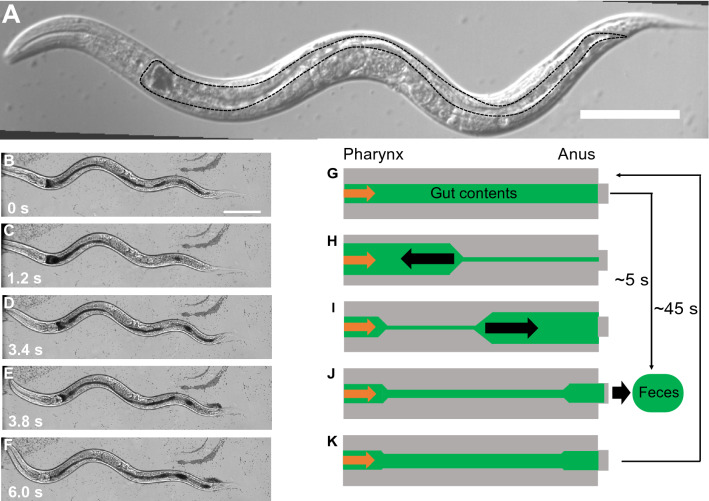
Body structures and intestinal functions of *C. elegans*. (**A**) DIC image of the body of *C. elegans*. The green area surrounded by a dotted line is the intestine. (**B-F**) DIC images of defecation. Black objects are particles stored in the intestine. Time is shown at lower left. **(G-K**) Defecation behavior of *C. elegans*. (**G**) The gut contents are distributed evenly before defecation; food materials are provided from the pharynx. Orange arrow indicates inlet from the pharynx. (**H**) When defecation begins, the gut contents flow toward the pharynx and accumulate in the anterior intestine. (**I**) Gut contents flow toward the anus and accumulate in the posterior intestine. (**J**) A portion of the gut contents is expelled through the anus. (**K**) Inlet from the pharynx and flow caused by body bending confer an even gut-content distribution, as shown in (**G**). Scale bars, 100 µm for (**A**) and (**B**).

As a model organism, *Caenorhabditis elegans* has been used for studies of food digestion and nutrient absorption^21–25^. *C. elegans* is a small (approximately 1-mm-long) multicellular terrestrial nematode and a filter feeder^21,26–28^. *C. elegans* worms have several advantages for biological and biomedical research. Their small body size and short life span (approximately 3 weeks) facilitate their maintenance^28^. The *C. elegans* genome has 60–80% homology with the human genome, despite the invertebrate nature of the animal^29,30^. The transparent *C. elegans* body allows visualization of tissues and cells by microscopy^28^. The only glucose transporter identified in *C. elegans* is FGT-1^25^, a homolog of the mammalian GLUT-2, which mediates transmembrane glucose transport by concentration gradient-mediated passive diffusion^31^. FGT-1 transporters are located on the basolateral membrane of intestinal cells facing the pseudocoel^25^. Although the glucose transporters that mediate glucose transport into intestinal cells from the intestinal lumen have not been identified in *C. elegans*^25^, whose transports are performed by SGLT in humans^31^, those transporters are considered to mediate glucose in *C .elegans* like SGLT. Therefore, the homology of genes and glucose transporters between humans and *C. elegans* makes this animal useful for experimental studies of intestinal nutrient uptake. In *C. elegans*, food bacteria ingested by the pharynx are digested and absorbed by microvilli in the intestinal lumen^32–34^ (Fig. 1A). Subsequently, *C. elegans* executes defecation by the periodic action of a stereotyped motor program that involves contractions of three sets of muscles; this program is regarded as the defecation motor program (DMP)^35^. The DMP functions as follows^36^ (Fig. 1B–K). The DMP begins with contractions of body wall muscles near the tail, which causes retrograde flow of gut contents toward the pharynx (Fig. 1G, H). Next, while the posterior body wall muscles relax, the anterior body wall muscles contract, causing anterograde flow of gut contents toward the anus (Fig. 1I). Finally, while the anterior body wall muscles relax, the muscles that open the anus contract, expelling through the anus some of the gut contents as feces (Fig. 1J). The total duration of the DMP is approximately 5 s; it is repeated at intervals of 45 ± 3 s (mean ± standard deviation) in healthy wild-type worms^35,36^. Because digestion and nutrient uptake associated with microbiota in *C. elegans* has been evaluated as a model experiment of pathogenic bacteria on symbiotic microbiota^37^, the mechanical role of DMP might have contributions involving nutrient uptake and host health^38^.

In *C. elegans*, tracer particles pass through the intestine in 3–10 min^21^. The mean gut residence time is reportedly < 2 min; approximately 43 ± 10% of the maximum volume of the intestinal lumen is expelled through the anus during each activation of the DMP^22^. The estimated time constant needed for digestion of bacteria is 14 ± 4 s^24^. We previously reported that a *C. elegans* worm must eat at least three bacterial cells per second to survive for 3 days, which implies the consumption of hundreds of thousands of bacteria daily^39^. The intestinal flows caused by the DMP have been proposed to facilitate nutrient uptake by intestinal cells^23^, but the mechanical relationship between gut-content movements and nutrient uptake in the intestine has not been elucidated. Therefore, the effects of intestinal flows on nutrient uptake by intestinal cells are unclear.

Here, we used fluorescent glucose and scaling analysis to investigate the effects of intestinal flows on glucose uptake by intestinal cells in *C. elegans*. We employed fluorescence microscopy to measure the distribution and amount of fluorescent glucose entering intestinal cells. We also used a high-speed camera and differential interference contrast (DIC) microscopy to observe intestinal flows caused by the DMP. Finally, we quantitatively evaluated the effects of intestinal flows on glucose uptake in the intestine.

## Results

### Distribution and uptake of glucose in intestinal cells

To evaluate the effects of intestinal flows on glucose uptake by intestinal cells, three defecation-defective mutants were used: *unc-16*(*e109*), which was isolated in genetic screens for Unc mutants^35^ and is defective in contractions of body wall muscles around the anterior intestine, and therefore the anterograde flow caused by the DMP is slow and weak; *egl-8*(*sa47*), which was isolated in genetic screens for DMP mutants and is defective in contractions of body wall muscles around the posterior intestine, and thus the retrograde flow caused by the DMP is slow and weak; and *exp-1*(*sa6*), which was isolated in genetic screens for DMP mutants and is defective in contractions of anus muscles, and therefore feces are expelled approximately once every six DMP cycles^35^.

**Figure 2:**
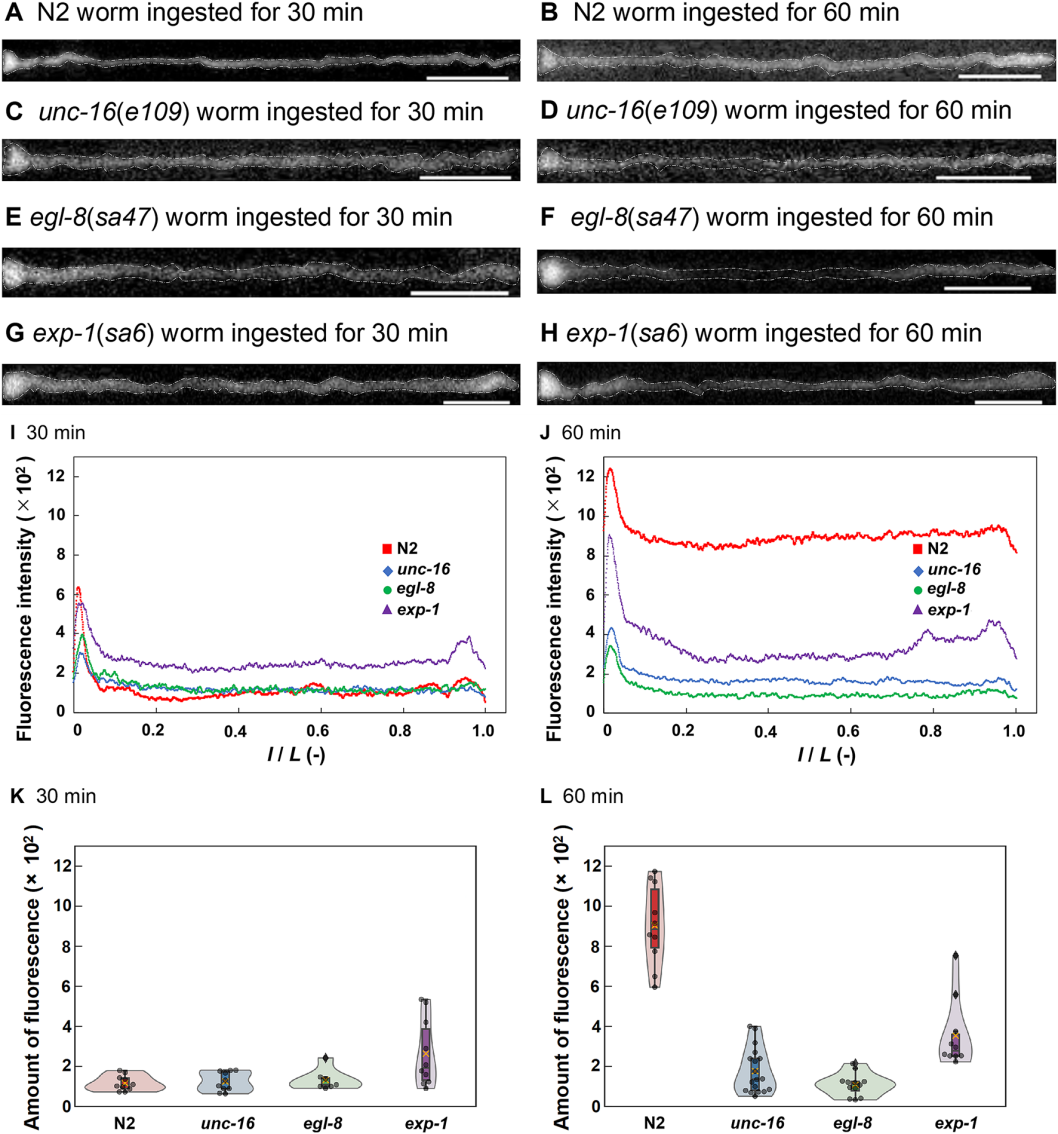
Distributions of fluorescent glucose in the intestine at 30 and 60 min. (**A-H**) Fluorescence micrographs of *C. elegans* intestine containing a 0.13% (v/v) suspension of OP50-1 labeled with fluorescent glucose. Left and right edges indicate entrance and exit, respectively, of the intestine. Scale bars, 100 µm. (**A**) N2 at 30 min. Broken line shows the outline of the intestine. (**B**) N2 at 60 min. (**C**) *unc-16*(*e109*) at 30 min. (**D**) *unc-16*(*e109*) at 60 min. (**E**) *egl-8*(*sa47*) at 30 min. (**F**) *egl-8*(*sa47*) at 60 min. (**G**) *exp-1*(*sa6*) at 30 min. (**H**) *exp-1*(*sa6*) at 60 min. (**I, J**) Distributions of 0.13% (v/v) OP50-1 labeled with fluorescent glucose in the intestine at 30 and 60 min. Colored dots show mean values. Vertical axis represents glucose fluorescence intensity in intestinal cells. *l/L* shows the position from the edge of the anterior part of the intestine divided by the length of the intestine. (**I**) 30 min; *n* ≥ 7 (each strain). (**J**) 60 min; *n* ≥ 7 (each strain). (**K, L**) Violin plots of glucose uptake per unit length of intestine. (**K**) 30 min. *n* ≥ 7 (each strain). (**L**) 60 min. *n* ≥ 7 (each strain). Data points and a box plot are shown in each violin plot. Cross plots and center lines in the box plots denote mean and median values, respectively.

First, we measured the distribution of glucose in intestinal cells using fluorescent glucose and a high-sensitivity camera. The wild-type strain N2 and the defecation-defective mutants *unc-16*, *egl-8*, and *exp-1* accumulated 0.13% (v/v) *Escherichia coli* OP50-1 labeled with fluorescent glucose in the intestines after 30 and 60 min (Fig. 2A–H). In the N2 intestine, the fluorescence intensity was highest in the most anterior part of the intestine; the intensities were similar among other parts (Fig. 2A, B). In *unc-16* and *egl-8* intestines, the glucose distribution was similar to the distribution in N2 (Fig. 2C–F). In the *exp-1* intestine, the fluorescence intensity was higher at both ends than in other parts (Fig. 2G, H).

Next, to quantify the effects of intestinal flows caused by the DMP on glucose uptake by intestinal cells, we analyzed fluorescence images of the intestine using ImageJ software (Fig. 2I, J). To measure glucose fluorescence in intestinal cells, the fluorescence intensity in the intestinal lumen was subtracted from the total fluorescence in the intestine. At 30 min, the fluorescence intensity was highest in *exp-1*; the fluorescence intensity in N2 was similar to the intensities in *unc-16* and *egl-8* (Fig. 2I). In contrast, at 60 min, glucose fluorescence was higher in N2 than in the mutants (Fig. 2J). Moreover, we calculated the amount of glucose uptake per unit length of the intestine by applying the trapezoidal rule to plotted curves of the fluorescence of glucose in each image (Fig. 2K, L). At 30 min, there was no difference in intestinal glucose uptake between N2 and *unc-16* or *egl-8* (Fig. 2K). At 60 min, although glucose uptake by the three mutants decreased or was unchanged, glucose uptake by N2 increased and became higher than uptake by the mutants (Fig. 2L).

### Accumulation of fluorescent particles in the intestine

**Figure 3:**
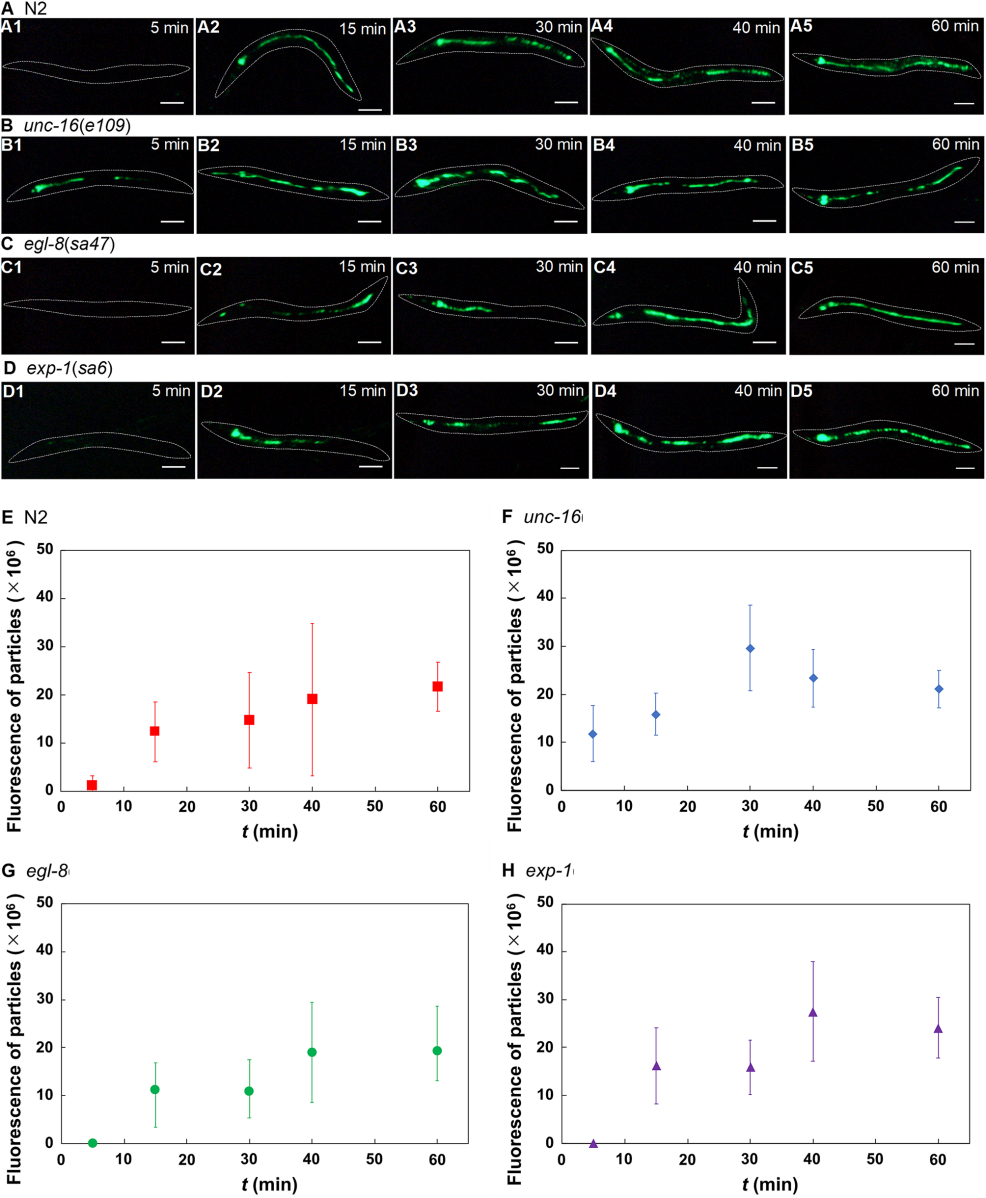
Distribution of fluorescent particles in the intestine at 5, 15, 30, 40, and 60 min. (**A-D**) Fluorescence images of particles in the intestine. White dotted lines are worm outlines. Time required for ingestion of particles is shown at upper right. Scale bars, 100 µm. (**A**) N2. **(B**) *unc-16*(*e109*). (**C**) *egl-8*(*sa47*). (**D**) *exp-1*(sa6). (**E–H**) Change in the total fluorescence of ingested particles in the intestine over time. Data are means ± standard deviations. (**E**) N2; *n* ≥ 12 worms. (**F**) *unc-16*; *n* ≥ 11 worms. (**G**) *exp-8*; *n* ≥ 10 worms. (**H**) *exp-1*; *n* ≥ 10 worms.

We next measured the fluorescence intensity of 0.5-μm-diameter fluorescent particles in the intestine after feeding for 5, 15, 30, 40, and 60 min. At 5 min, fluorescence was observed only in *unc-16* (Fig. 3A1, B1, C1, D1). At 15 min, fluorescence was distributed throughout the intestine in N2 and *unc-16*, while it was localized to the anterior or posterior part in *exp-1* and *egl-8* (Fig. 3A2, B2, C2, D2). At 30 min, the particles were distributed throughout the intestine, except in *egl-8* (Fig. 3A3, B3, C3, D3). At 40 and 60 min, the particles were distributed throughout the intestine in all strains (Fig. 3A4, A5, B4, B5, C4, C5, D4, D5). Although the timing and rate of increase in particle accumulation differed among strains, the total fluorescence of accumulated particles in the worm was similar among the four strains (Fig. 3E–H).

### Intestinal flows during the DMP

**Figure 4:**
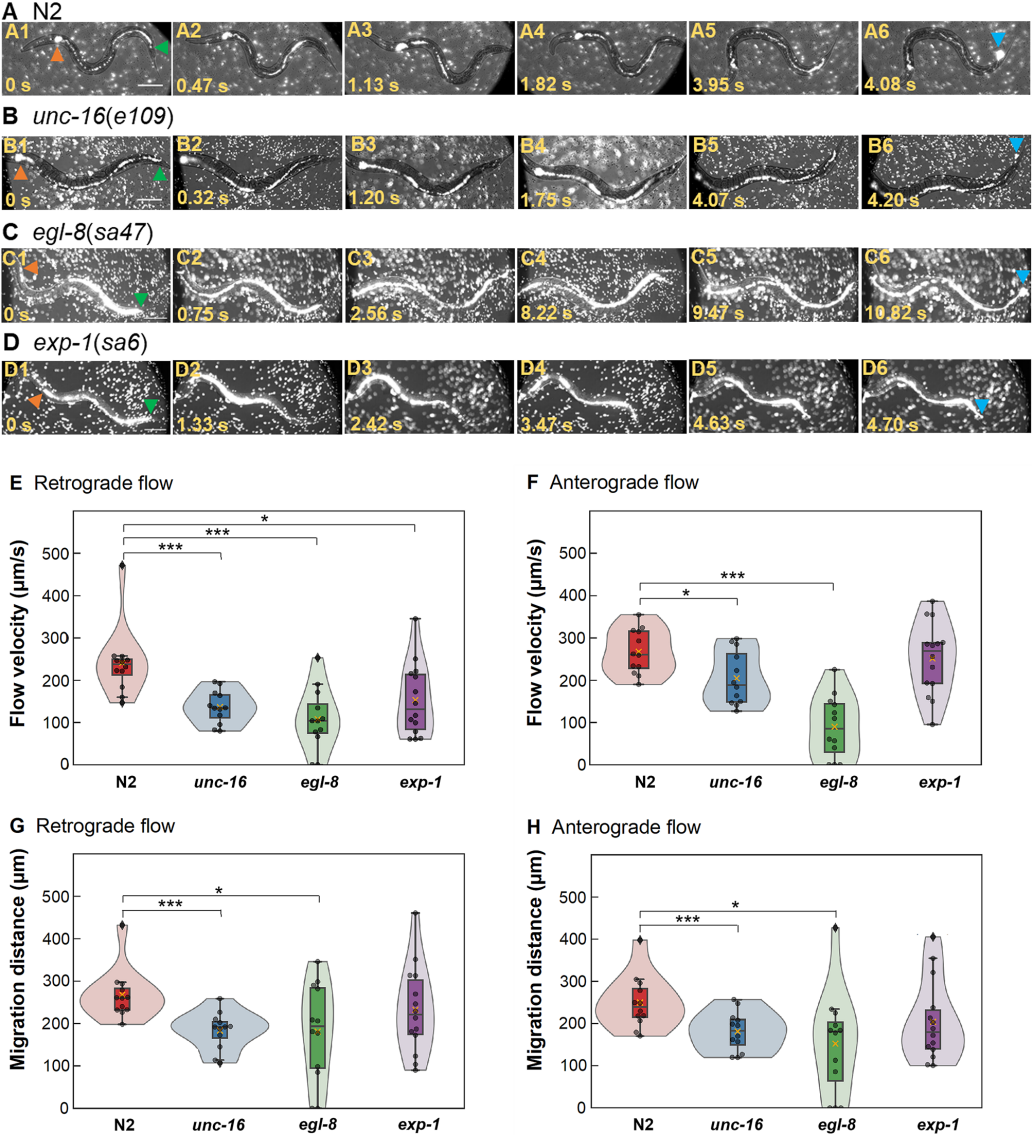
Flows of gut contents during defecation. **(A–D)** Flow of fluorescent particles (white dots) in the intestine during the DMP. Orange and green arrowheads indicate edges of the anterior and posterior parts of the intestine, respectively; light-blue arrowheads indicate feces expelled from the anus. Elapsed time is shown at bottom left. Scale bar, 100 µm. (**A**) N2. (**B**) *unc-16*(*e109*). (**C**) *egl-8*(*sa47*). (**D**) *exp-1*(sa6). (**E, F**) Violin plots of particle retrograde and anterograde flow velocities in the intestine caused by DMP. (**E**) Retrograde flow; *n* ≥ 12 (each strain). (**F**) Anterograde flow; *n* ≥ 12 (each strain). (**G, F**) Violin plots of particle migration distances in the intestine by DMP-induced retrograde or anterograde flow. (**G**) Retrograde flow; *n* ≥ 12 (each strain). (**H**) Anterograde flow; *n* ≥ 12 (each strain). Data points and a box plot are shown in each violin plot. Cross plots and center lines in box plots denote mean and median values, respectively. Significant differences are indicated by *P < 0.05 and ***P < 0.005 according to Student’s *t*-test or Welch’s *t*-test.

We next visualized intestinal flows during the DMP by DIC microscopy (Fig. 4A–D, see Supplementary moves). In the N2 intestine, when the DMP began, the particles in the posterior part of the intestine experienced retrograde flow caused by the DMP; thus, they were stored in the anterior part (Fig. 4A1–A3). Next, they were transported to the anus through anterograde flow caused by the DMP; they were stored in the posterior part of the intestine (Fig. 4A4, A5). Finally, some particles in the posterior part of the intestine were expelled through the anus (Fig. 4A6). In the *unc-16* intestine, when the DMP began, particles in the posterior part of the intestine experienced retrograde flow and were stored in the anterior part and first half of the posterior part of the intestine (Fig. 4B1–B3). Next, these stored particles were transported to the anus through anterograde flow and stored in the posterior part of the intestine (Fig. 4B4, B5). Finally, some particles in the posterior part of the intestine were expelled to the outside of the body (Fig. 4B6). In the *egl-8* intestine, when the DMP began, particles in the first half of the posterior part of the intestine experienced retrograde flow and were stored in the anterior part of the intestine (Fig. 4C1–C3). Next, they were transported to the anus through anterograde flow and were stored in the posterior part of the intestine (Fig. 4C4, C5). Finally, some particles in the posterior part of the intestine were expelled through the anus (Fig. 4C6). In the *exp-1* intestine, the particle flows were similar to the flows in N2 (Fig. 4D). Therefore, intestinal flows during the DMP differed according to whether the DMP was defective and the nature of any defect.

To quantify the differences in intestinal flows during the DMP, we measured the flow velocities of particles flowing from the posterior to the anterior part of the intestine by DMP-induced retrograde flow, as well as the flow velocities of particles flowing from the anterior to the posterior part of the intestine through anterograde flow; we also measured the migration distances of the group of particles. There was no significant difference between N2 and *exp-1* in terms of anterograde flow velocity (*P* > 0.05 by Welch’s *t*-test) (Fig. 4E, F). Therefore, anterograde flow velocity is not dependent on the ability to expel gut contents. However, the anterograde flow velocity differed between N2 and *unc-16* (Student’s *t*-test), retrograde flow velocity differed between N2 and *egl-8* (Welch’s *t*-test) and between N2 and *exp-1*(Welch’s *t*-test), and anterograde flow differed between N2 and *unc-16* (Welch’s *t*-test) and between N2 and *egl-8* (Welch’s *t*-test). Thus, intestinal flow velocities during the DMP differed according to defects in retrograde and anterograde flow caused by the DMP. Furthermore, the retrograde and anterograde flow velocities were significantly higher in N2 than in *unc-16* or *egl-8*.

There was no significant difference between N2 and *exp-1* in terms of migration distance by retrograde or anterograde flow (*P* > 0.05; Welch’s *t*-test or Student’s *t*-test) (Fig. 4G, H). Therefore, migration distances by retrograde and anterograde flow do not depend on the ability to expel gut contents. However, migration distances by retrograde and anterograde flow significantly differed between N2 and *unc-16* (Welch’s *t*-test), while migration distances by retrograde and anterograde flow significantly differed between N2 and *egl-8* (Welch’s or Student’s *t*-test). Thus, retrograde or anterograde particle migration distance caused by the DMP was dependent on the presence of a functional retrograde or anterograde flow mechanism. Additionally, the gut content migration distances by retrograde and anterograde flows were significantly greater in N2 than in *unc-16* or *egl-8*.

### Relationship between glucose uptake and intestinal flows

**Figure 5:**
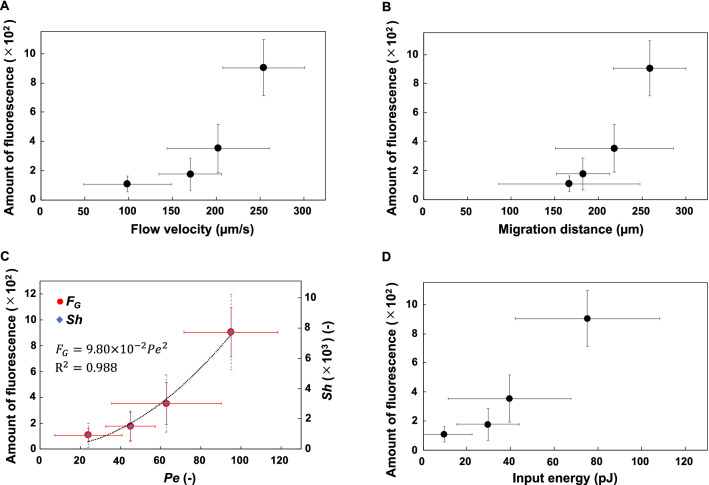
Associations of glucose fluorescence in intestinal cells after ingestion of 0.13% (v/v) OP50-1 labeled with fluorescent glucose for 60 min with intestinal flows during defecation. (**A**) Associations of glucose uptake in intestinal cells with mean particle retrograde and anterograde flow velocities. (**B**) Associations of glucose uptake in intestinal cells with mean particle retrograde and anterograde flow velocities. (**C**) Associations of glucose uptake or Sherwood number (Eq. [4]) with Péclet number (Eq. [1]). Fluorescence data were fitted to the function *F*_*G*_ = 9.80 × 10^−2^*Pe*^*2*^ with R^2^ = 0.988. (**D**) Associations of glucose uptake at 60 min with input energy during the DMP (Eq. [8]). Error bars indicate standard deviations.

We next evaluated the relationship between glucose uptake and flow velocity or migration distance. There was an association between glucose uptake by intestinal cells at 60 min and the mean intestinal flow velocity (Fig. 5A). Glucose uptake tended to increase with increasing flow velocity. There was an association between glucose uptake by intestinal cells at 60 min and the mean particle migration distance (Fig. 5B). Therefore, glucose uptake increased with increasing migration distance.

To quantify the effects of DMP-induced intestinal flows on glucose uptake by intestinal cells, we used two dimensionless numbers: the Péclet number (*Pe*) and the Sherwood number (Sh)^40,41^. *Pe* is defined as the ratio of transport caused by advection (*i*.*e*., flow velocity) to transport caused by molecular diffusion. *Pe* is given by:

1 $$ \begin{array}{*{20}c} {Pe = \frac{UL}{D},} \\ \end{array} $$

where *U* is the characteristic velocity, *L* is the characteristic length, and *D* is the diffusion coefficient. In the *C. elegans* intestine, *D* can be estimated^42^ as 0.69 × 10^−9^ m^2^/s (*i*.*e*., the diffusion coefficient of glucose in water at 25 °C). *UL* is calculated as the mean of the product of migration velocity and distances of particles in retrograde and anterograde flow. The *Pe* values were 95.2 for N2, 45.0 for *unc-16*, 24.0 for *egl-8*, and 62.9 for *exp-1*. There was an association between *Pe* and glucose uptake by intestinal cells (*F*_*G*_) at 60 min (Fig. 5C). Therefore, *F*_*G*_ increases in proportion to the square of *Pe* (*F*_*G*_ = 9.80 × 10^−2^*Pe*^*2*^).

*Sh* is defined at an interface as the ratio of the mass transfer rate by advection and diffusion relative to the mass transfer rate by diffusion alone. *Sh* is given by:

2 $$ \begin{array}{*{20}c} {Sh = \frac{{U_{m} L}}{D},} \\ \end{array} $$

where *U*_*m*_ is the permeation velocity at the interface, *L* is the characteristic length, and *D* is the diffusion coefficient. By assuming that mass absorption occurs on the inner surface of a circular tube of diameter *R* and length *L*, *U*_*m*_ (averaged over the surface *πRL*) can be expressed as:

3 $$ \begin{array}{*{20}c} {U_{m} = \frac{{m_{s} }}{\pi RL},} \\ \end{array} $$

where *m*_*s*_ is the mass flow velocity through the surface. In this study, *m*_*s*_ was the amount of glucose taken up by intestinal cells per unit time; it was assumed to be proportional to the fluorescence intensity of glucose in intestinal cells (*F*_*G*_). *R* was determined to be 15 µm in this study and a previous study^43^.

By combining (2) and (3), and using the relations $m_{s} \propto F_{G} \propto Pe^{2}$ under constant *R* and *D* conditions, *Sh* can be rewritten as:

4 $$ \begin{array}{*{20}c} {Sh = \frac{{m_{s} }}{\pi RD} \propto F_{G} \propto Pe^{2} .} \\ \end{array} $$

In Fig. 5C, *Sh* is plotted on the vertical axis; it is proportional to the square of *Pe*.

To quantify DMP energetics, we estimated the energy input required to pump the gut contents during the DMP. The input energy *E* can be derived by applying Darcy-Weisbach’s formula:

5 $$ \begin{array}{*{20}c} {E = PQT,} \\ \end{array} $$

where *P* is the pressure drop in the intestinal lumen, *Q* is the flow rate, and *T* is the duration of retrograde or anterograde flow during the DMP*.* By assuming that the intestine is a straight tube of circular cross-section and that the Hagen–Poiseuille law is applicable^39,44^, *P* is given by:

6 $$ \begin{array}{*{20}c} {P = \frac{64}{{\text{Re}}} \frac{{L_{m} }}{R} \frac{{\rho U^{2} }}{2} = \frac{{32\mu L_{m} U}}{{R^{2} }},} \\ \end{array} $$

where *Re* is the Reynolds number, *L*_*m*_ is the particle migration distance by DMP-induced retrograde or anterograde flow, *R* is the inner diameter of the intestinal lumen, *U* is the velocity of retrograde or anterograde flow during the DMP, *ρ* is the density of the gut contents, and *μ* is the viscosity of the gut contents. Thus, *Q* is given by:

7 $$ \begin{array}{*{20}c} {Q = \frac{\pi }{4}R^{2} U.} \\ \end{array} $$

Therefore, Eq. (5) can be rewritten as:

8 $$ \begin{array}{*{20}c} {E = 8\pi \mu L_{m} U^{2} T.} \\ \end{array} $$

There was an association between the glucose fluorescence intensity in intestinal cells at 60 min, *F*_*G*_, and the input energy $E$ during the DMP obtained from Eq. (8) (Fig. 5D). Thus, the amount of glucose taken up tended to increase with increasing energy input.

## Discussion

We investigated glucose uptake in the *C. elegans* intestine. When using a high glucose concentration 1.3% (v/v), we found no significant difference in glucose uptake among the four strains (see Supplementary Information). The lack of a difference in glucose uptake between the wild-type and mutant strains is likely because of saturated glucose fluorescence. In contrast, when using glucose at 0.13% (v/v), glucose uptake at 60 min was considerably higher in N2 than in the mutants (Fig. 2J, L). Therefore, the difference in glucose uptake between N2 and the mutants might be caused by differences in the amounts of gut contents or in the characteristics of DMP-induced intestinal flow. Note that at a glucose concentration of 0.13%, hyperglycemia did not appear to have a significant effect on the regulation of glucose uptake by worms. This is because the worms have the capacity to absorb more glucose, since they absorbed nearly three times as much glucose at a glucose concentration of 1.3%.

At 40 and 60 min, there was minimal variation in the amounts of gut contents among N2 and the mutants (Fig. 3E–H). This suggests that a defective DMP does not affect the amounts of gut contents. Thus, the differences in glucose uptake between N2 and the three mutants were not caused by differences in the amounts of gut contents.

Next, we analyzed the intestinal flows induced by the DMP (Fig. 4). The gut retrograde- and anterograde-flow velocities were significantly higher in N2 than in *unc-16* or *egl-8* (Fig. 4E, F). The retrograde and anterograde migration distances of the gut contents were significantly greater in N2 than in *unc-16* or *egl-8* (Fig. 4G, H). Moreover, glucose uptake by intestinal cells tended to increase with increasing flow velocities or migration distances (Fig. 5A, B). Therefore, the difference in glucose uptake between N2 and the three mutants resulted from different DMP-induced intestinal flow characteristics.

Glucose uptake increased with the square of *Pe* (*F*_*G*_ = 9.80 × 10^−2^*Pe*^2^) (Fig. 5C). Importantly, mass transport into a wall induced by flow in a tube with peristalsis increases with the square of *Pe* in a large *Pe* regime^45^ (see Supplementary Information). A similar scaling (*i.e.,* Taylor dispersion) has been reported^46^, in which apparent diffusivity in a tube is enhanced by flow with a factor of *Pe*^2^. Considering that *F*_*G*_ and *Sh* both depend on *Pe*, the DMP-induced intestinal flows significantly promote glucose uptake by intestinal cells, compared with molecular diffusion alone.

Finally, glucose uptake increased with increasing energy input (Fig. 5D). Moreover, we previously reported that the energy obtained by digestion of a single OP50-1 bacterium was 7.0 × 10^−7^ kcal (1 kcal = 4.184 kJ); the number of bacteria required to survive for 3 days was approximately three cells per second^39^. Using these values, we estimate the minimum calorie intake of a worm in 1 h to be 31.6 J. Therefore, minimal energy (in the order of picojoules) was used for glucose uptake compared to the energy obtained from ingested bacteria. Because glucose uptake increased with increasing intestinal flow velocities, N2 likely had the greatest capacity for glucose uptake among the four strains.

In summary, glucose uptake by intestinal cells was higher in N2 than in *unc-16*, *egl-8*, or *exp-1*. Glucose uptake tended to increase increasing intestinal flow velocities during the DMP. Glucose uptake and the Sherwood number increased with the square of *Pe*. Glucose uptake also tended to increase with increasing energy input, despite far greater calorie intake. We conclude that *C. elegans* exploits DMP-induced intestinal flows to promote glucose uptake by intestinal cells, which may be the primary function of the DMP.

## Materials and methods

### Worm preparation

The wild-type strain N2 was obtained from Dr. Shohei Mitani (Tokyo Women’s Medical University School of Medicine, Tokyo, Japan). The mutant strains *unc-16*(*e109*), *exp-1*(*sa6*), and *egl-8*(*sa47*) were obtained from the *Caenorhabditis* Genetics Center (University of Minnesota, Minneapolis, MI, USA), and had been used without further outcrossing to the wild-type strain. The worms were maintained on nematode growth medium plates at 20 °C using standard protocols^26,28^. Worms were removed from nematode growth medium by washing with 700 µL of M9 buffer^28^. OP50-1 cells were cultured in liquid Luria–Bertani (LB) medium in an incubator at 37 °C with shaking at 200 rpm for 12–14 h^28^.

### Fluorescence imaging

To image the intestine, we used red fluorescent glucose (Glucose Uptake Assay Kit-Red; excitation wavelength, 560 nm; fluorescence wavelength, 572 nm; Dojindo Laboratories, Japan) to label OP50-1 cells in accordance with the manufacturer’s protocol^47,48^. Bacteria were washed three times with 2 mL of phosphate-buffered saline (pH 7.4) for 10 min at 2000 g in a centrifuge (EX-126, Tomy Seiko Co., Ltd., Japan). The washed cells were stored for 60 min at 37 °C and 200 rpm in an incubator (CN-25C, Mitsubishi Electric Corporation, Japan). The cells were labeled with a 100-fold dilution of fluorescent glucose for 30 min at 37 °C and 200 rpm. Finally, the fluorescent cells were washed three times with 2 mL of phosphate-buffered saline (pH 7.4) for 10 min at 2000 g in a centrifuge.

### Fluorescence imaging of glucose in intestinal cells

To image glucose in the intestinal cells, 5–10 L4 or young adult worms were fed 1.3% or 0.13% (v/v) OP50-1 labeled with fluorescent glucose in 200 µL of M9 buffer in a 14-mm-diameter glass-bottom dish (Matsunami, Japan) for 5, 15, 30, or 60 min at 20 °C. After feeding, the worms were washed twice with 100 µL of M9 buffer. Washed worms were transferred to 3–4 μL of M9 buffer on a 2% (w/v) agarose gel on a microscope slide (surface area, 24 × 60 mm^2^; thickness, 0.12–0.17 mm; Matsunami) and covered with a coverslip (surface area, 24 × 24 mm^2^; thickness, 0.17 mm; Matsunami) to minimize dehydration. Fluorescence was visualized using a fluorescence microscope equipped with a digital camera (DP73, Olympus, Japan) or a high sensitivity camera (DU-897E-CS0-#BV, Andor Technology Ltd., UK), a 10 × dry objective lens (UPlanSApo 10 × l NA, 0.40; WD, 3.1 mm; Olympus), and a mirror (U-MRFPHQ; excitation, 535–555 nm; emission, 570–625 nm; Olympus). Glucose fluorescence in the worm was analyzed using ImageJ software^49^. We measured the distributions of 0.13% (v/v) OP50-1 labeled with fluorescent glucose in the intestine at 30 and 60 min, respectively. Note that the intestinal lumen was manually distinguished using the brightness gap between the lumen and the intestinal cells. The glucose uptake was evaluated by subtracting the total fluorescence intensity in the lumen from that in the whole worm. The background noises in the image were initially subtracted.

### Quantification of gut contents

To quantify particles in the intestine, 10–15 young adult worms were fed 0.13% (v/v) fluorescent particles of 0.5 μm diameter (excitation wavelength, 505 nm; fluorescence wavelength, 514 nm; Thermo Fisher Scientific) mixed with 2% (v/v) OP50-1 in 200 μL of M9 buffer or the MC solutions in a 14-mm-diameter glass-bottom dish (Matsunami) for 5, 15, 30, 40, and 60 min at 20 °C. Next, worms were fixed in 200 μL of 99% ethanol (Fujifilm Wako Pure Chemical Corporation, Japan) for 1 min and washed three times in 1 mL of M9 buffer, then centrifuged for 10 min at 1413 g (3220, Kubota Corporation, Japan) to remove excess particles. Finally, the worms were transferred to 100 μL of M9 buffer in a 14-mm-diameter glass-bottom dish (Matsunami). Particle fluorescence was observed using a fluorescence microscope with a 4 × dry objective lens (UPlanFLN 4 × ; NA, 0.13; WD, 17 mm; Olympus). The total fluorescence of the ingested particles was analyzed using ImageJ software^39^.

### Visualization of intestinal flows

L4 or young adult worms (*n* = 5–10) were transferred to 200 μL of M9 buffer containing 0.013% (v/v) fluorescent particles (diameter, 1.0 µm; excitation wavelength, 505 nm; fluorescence wavelength, 514 nm; Thermo Fisher Scientific) mixed with 2% (v/v) OP50-1 in a chamber (surface area, 9 × 9 mm^2^; Bio-Rad Laboratories) on a microscope slide (surface area, 24 × 60 mm^2^; thickness, 0.12–0.17 mm; Matsunami). The suspension was covered by a coverslip (surface area, 24 × 24 mm^2^; thickness, 0.17 mm; Matsunami) to minimize dehydration. Particle movement in the intestine during the DMP was observed using an upright DIC microscope (BX51WI, Olympus) with a high-speed camera (SA3, Photron, Japan), a 20 × dry objective lens (UPlanSApo 20 × ; NA, 0.75; WD, 0.6 mm; Olympus), a halogen lamp (U-LH100HGAPO, Olympus), a power supply unit (U-RFL-T, Olympus), and a mirror (excitation, 479–495 nm; emission, 510–550 nm; U-MWIBA3, Olympus)^39,50^. High-speed image sequences of approximately 45 s duration at 60 fps were recorded using a high-speed video camera (see Supplementary moves). We manually traced the positions of the particles in the intestine during the DMP using ImageJ software^49^. From the frame numbers and pixel numbers, we calculated the time-averaged particle flow velocities and the mean time-averaged particle velocity through division of the particle migration distance by the number of frames.

### Statistical analysis

Statistical analysis was performed using Microsoft Excel 2019 (Microsoft Corporation, Redmond, WA, USA) on macOS Sierra (Apple Inc., Cupertino, CA, USA). A value of *n* indicates the number of the worms, not the number of particles tracked in total. A value of *p* < 0.05 was considered to indicate statistical significance.

## Supplementary Information

Supplementary Video 1.

Supplementary Video 2.

Supplementary Video 3.

Supplementary Video 4.

Supplementary Information 1.

## Data Availability

The data that support the findings of this paper are available from the corresponding author upon request. Supplementary information is provided with the paper to support the experimental results.
