# Supplementary material for: Reciprocating intestinal flows enhance glucose uptake in C. elegans
Source: Sci Rep. 2022 Sep 21;12:15310. doi: 10.1038/s41598-022-18968-1 (PMC9492717; doi:10.1038/s41598-022-18968-1)
Supplement: Supplementary file 5 — Supplementary Information 1. [file 41598_2022_18968_MOESM5_ESM.docx]

**Reciprocating intestinal flows enhance glucose uptake in *C. elegans***

**Supplementary Information**

**1. Distribution and intake of glucose in intestinal cells at high nutrient concentrations**

We evaluated glucose uptake by intestinal cells using fluorescent glucose. Supplementary Figure 1A-D shows that the intestines of N2, *unc-16*(*e109*), *egl-8*(*sa47*), and *exp-1*(*sa6*) accumulated 1.3% (v/v) *E. coli* OP50-1 labeled with fluorescent glucose after 5, 15, 30, and 60 min. In the N2 intestine, the fluorescence intensity was highest in the most anterior part; the intensities were similar among other parts (Supplementary Fig. 1A). In the *unc-16*(*e109*) intestine, fluorescence intensity was lowest in the posterior part up to 5 min; fluorescence distribution was similar to the distribution in N2 after 30 min (Supplementary Fig. 1B). In the *egl-8*(*sa47*) intestine, fluorescence intensity was lowest in the posterior intestine up to 15 min; fluorescence distribution was similar to the distribution in N2 after 30 min (Supplementary Fig. 1C). In the *exp-1*(*sa6*) intestine, the fluorescence distribution was identical to the distribution in N2 (Supplementary Fig. 1D).

Next, we analyzed the intestinal fluorescence distribution after feeding for 5, 15, 30, and 60 min using ImageJ software (Supplementary Fig. 2). Although the fluorescence values in N2 were similar to the values in *unc-16*(*e109*) and *exp-1*(*sa6*), fluorescence values were lower in *egl-8*(*sa47*) up to 15 min (Supplementary Fig. 2A, B). The fluorescence values of the four strains were similar at 30 and 60 min in each part of the intestine (Supplementary Fig. 2C, D). Moreover, we calculated glucose uptake per unit length of the intestine by applying the trapezoidal rule to glucose fluorescence curves (Supplementary Fig. 2E-H). At 5, 15, 30, and 60 min, glucose uptake by intestinal cells was similar among the four strains.

**Supplementary Figure 1.** Fluorescence micrographs of the *C. elegans* intestine containing a 1.3% (v/v) suspension of OP50-1 labeled with fluorescent glucose at 5, 15, 30, and 60 min. The entrance and exit of the intestine are at left and right, respectively. Scale bars, 100 µm. (**A1**) N2 after 5 min. (**A2**) N2 after 15 min. (**A3**) N2 after 30 min. (**A4**) N2 after 60 min. (**B1**) *unc-16*(*e109*) after 5 min. (**B5**) *unc-16*(*e109*) after 15 min. (**B3**) *unc-16*(*e109*) after 30 min. (**B4**) *unc-16*(*e109*) after 60 min. (**C1**) *egl-8*(*sa47*) after 5 min. (**C2**) *egl-8*(*sa47*) after 15 min. (**C3**) *egl-8*(*sa47*) after 30 min. (**C1**) *egl-8*(*sa47*) after 5 min. (**C2**) *egl-8*(*sa47*) after 15 min. (**C3**) *egl-8*(*sa47*) after 30 min. (**C4**) *egl-8*(*sa47*) after 60 min. (**D1**) *exp-1*(*sa6*) after 5 min. (**D2**) *exp-1*(*sa6*) after 15 min. (**D3**) *exp-1*(*sa6*) after 30 min. (**D4**) *exp-1*(*sa6*) after 60 min.

**
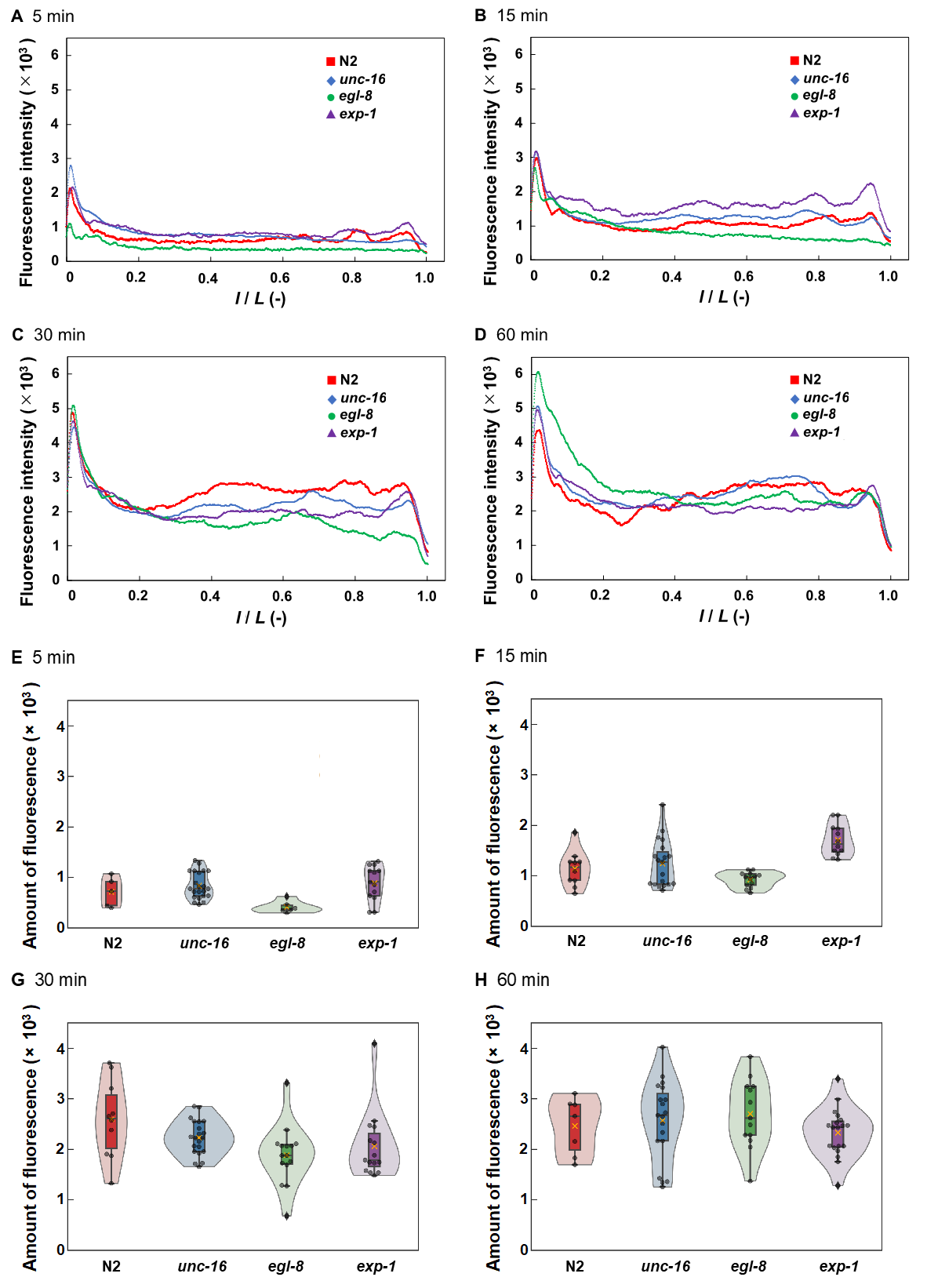
**

**Supplementary Figure 2.** (A-D) Distribution of 1.3% (v/v) OP50-1 labeled with fluorescent glucose in the intestine at 5, 15, 30, and 60 min in N2 and the three mutants. Colored dots show mean values. Vertical axis represents glucose fluorescence intensity. *l/L* shows the position from the edge of the anterior part of the intestine divided by the length of the intestine. (**A**) 5 min; *n* ≥ 5 (each strain). (**B**) 15 min; *n* ≥ 10 (each strain). (**C**) 30 min; *n* ≥ 10 (each strain). (**D**) 60 min; *n* ≥ 7 (each strain). (**E-H**) Violin plots of glucose uptake per unit length of the intestine. (**E**) 5 min; *n* ≥ 5 (each strain). (**F**) 15 min; *n* ≥ 7 (each strain). (**G**) 30 min; *n* ≥ 10 (each strain). (**H**) 60 min; *n* ≥ 10 (each strain). Data points and a box plot are shown in each violin plot. Cross plots and center lines in box plots show means and medians, respectively.

**2. Mass transport induced by peristalsis**

To validate our finding that glucose uptake increased with the square of *Pe* (*F_G_* = 9.80 × 10^-2^*Pe^2^*) (Fig. 5C), we compared solute dispersion between our study and a previous study^45^. In the prior work, mass transport equations were analytically derived by assuming Hagen–Poiseuille flow along a long, slender tube of radius *d*(*z*, *t*) and length *l* that experienced wall deformation by peristalsis. The absorption of solute on the wall of the tube was expressed by the following equation:

$$\begin{aligned} D\frac{\partial C}{\partial r}+\gamma C=0,\#\left( S1 \right) \end{aligned}$$

where *D* is the mass diffusion coefficient, *γ* is the surface absorption velocity, and *C*(*r*, *z*, *t*) is the cross-sectionally averaged solute concentration. *D* is promoted by a factor known as Taylor dispersion^46^. The mass diffusion coefficient *D* promoted by peristaltic flow-induced dispersion in the tube can be derived as^45^:

$$\begin{aligned} D_{eff}=D\left[ 1+{(\varphi ka)}^{2}\frac{{{Pe}_{wave}}^{2}}{48}+\frac{1}{2}\varphi^{2}\frac{6{{Pe}_{wave}}^{2}}{2{{Pe}_{wave}}^{2}+1}-\frac{1}{2}\varphi^{2}\frac{1}{2{{Pe}_{wave}}^{2}+1} \right],\#\left( S2 \right) \end{aligned}$$

where *φ* is the dimensionless amplitude of the cross-sectional area of the peristalsis wave, *k* is the wave number of the peristalsis wave, and *a* is the time averaged radius of the tube. Moreover, *Pe_wave_* is the Péclet number of the peristalsis wave^45^, defined as *Pe_wave_* = $\sqrt{2}\pi U_{wave}L_{wave}/D$, where $U_{wave}$ is the wave propagation velocity and $L_{wave}$ is the wave length. By assuming that $U_{wave}$ is equivalent to the mean retrograde and anterograde migration velocities of particles, and the migration distance of particles defines $L_{wave}$, we have *Pe_wave_* = $\sqrt{2}\pi$*Pe*. At a small *Pe_wave_*, the term containing *Pe_wave_*^-2^ is dominant in equation (S2). At a large *Pe_wave_*, the term containing *Pe_wave_*^2^ is dominant in equation (S2), indicating that Taylor dispersion is dominant with respect to flow-induced dispersion in the tube. Taylor dispersion is dominant^45^ at *Pe_wave_* ≥ 8/(*ka*). In the *C. elegans* intestine, *k* is the reciprocal of the migration distance and *a* is the radius of the lumen. Accordingly, *Pe_wave_* and 8/(*ka*) were 422.7 and 276.0 for N2, 200.0 and 194.5 for *unc-16*(*e109*), 106.7 and 177.7 for *egl-8*(*sa47*), and 279.6 and 232.7 for *exp-1*(*sa6*), respectively. Therefore, Taylor dispersion is dominant in the *C. elegans* intestine. This explains why glucose uptake in the *C. elegans* intestine could be expressed as the square of *Pe*.
